# A genome-wide association study for melatonin secretion

**DOI:** 10.1038/s41598-022-12084-w

**Published:** 2022-05-16

**Authors:** Pi-Hua Liu, Gwo-Tsann Chuang, Chia-Ni Hsiung, Wei-Shun Yang, Hsiao-Chia Ku, Yi-Ching Lin, Yi-Shun Chen, Yu-Yao Huang, Chia-Hung Lin, Wen-Yi Li, Jou-Wei Lin, Chih-Neng Hsu, Juey-Jen Hwang, Karen Chia-Wen Liao, Meng-Lun Hsieh, Hsiao-Lin Lee, Chen-Yang Shen, Yi-Cheng Chang

**Affiliations:** 1 Clinical Informatics and Medical Statistics Research Center, College of Medicine, Chang Gung University, Taoyüan, Taiwan; 2 Division of Endocrinology and Metabolism, Department of Internal Medicine, Chang Gung Memorial Hospital at Linkou, Taoyüan, Taiwan; 3 Department of Pediatrics, College of Medicine, National Taiwan University Hospital, National Taiwan University, Taipei, Taiwan; 4 Graduate Institute of Medical Genomics and Proteomics, National Taiwan University, 5F, No.2, Xuzhou Rd., Zhongzheng Dist., Taipei, 100 Taiwan; 5 Data Science Statistical Cooperation Center, Institute of Statistical Science, Academia Sinica, Taipei, Taiwan; 6 Department of Internal Medicine, National Taiwan University Hospital Hsin-Chu Branch, Hsinchu, Taiwan; 7 Department of Laboratory Medicine, National Taiwan University Hospital Hsin-Chu Branch, Hsinchu, Taiwan; 8 Department of Medical Nutrition Therapy, Lin-Kou Chang-Gung Memorial Hospital, Chang-Gung Medical Foundation, Taoyüan, Taiwan; 9 Department of Chinese Medicine, College of Medicine, Chang Gung University, Taoyüan, Taiwan; 10 Department of Internal Medicine, National Taiwan University Hospital Yunlin Branch, Yunlin, Taiwan; 11 Cardiovascular Center, National Taiwan University Hospital Yun-Lin Branch, Yunlin, Taiwan; 12 Department of Internal Medicine, National Taiwan University Hospital, Taipei, Taiwan; 13 Biological Sciences Division, University of Chicago, Chicago, IL 60637 USA; 14 Institute of Biomedical Sciences, Academia Sinica, Taipei, Taiwan; 15 College of Public Health, China Medical University, Taichung, Taiwan

**Keywords:** Genetics, Endocrinology

## Abstract

Melatonin exerts a wide range of effects among various tissues and organs. However, there is currently no study to investigate the genetic determinants of melatonin secretion. Here, we conducted a genome-wide association study (GWAS) for melatonin secretion using morning urine 6-hydroxymelatonin sulfate-to-creatinine ratio (UMCR). We initially enrolled 5000 participants from Taiwan Biobank in this study. After excluding individuals that did not have their urine collected in the morning, those who had history of neurological or psychiatric disorder, and those who failed to pass quality control, association of single nucleotide polymorphisms with log-transformed UMCR adjusted for age, sex and principal components of ancestry were analyzed. A second model additionally adjusted for estimated glomerular filtration rate (eGFR). A total of 2373 participants underwent the genome-wide analysis. Five candidate loci associated with log UMCR (*P* value ranging from 6.83 × 10^−7^ to 3.44 × 10^−6^) encompassing *ZFHX3*, *GALNT15*, *GALNT13*, *LDLRAD3* and intergenic between *SEPP1* and *FLJ32255* were identified. Similar results were yielded with further adjustment for eGFR. Interestingly, the identified genes are associated with circadian behavior, neuronal differentiation, motor disorders, anxiety, and neurodegenerative diseases. We conducted the first GWAS for melatonin secretion and identified five candidate genetic loci associated with melatonin level. Replication and functional studies are needed in the future.

## Introduction

Melatonin is a pleiotropic hormone primarily synthesized and secreted from the pineal gland. Many other tissues can also produce it, including leukocytes, bone marrow, gastrointestinal tract, neuronal cells, and gonads^1–3^. Melatonin regulates various physiological processes, including circadian and seasonal rhythms, energy and glucose metabolism, antioxidant effects, anti-inflammatory actions, and immune function^1,3–6^. There are many studies showing associations between melatonin and many disorders, including certain types of mental illness, cancer, cardiovascular disease, metabolic syndrome, type 2 diabetes, and obesity^6–11^. Melatonin is secreted into the circulation following a circadian rhythm with peak levels at night^12^. Aging was once thought to be directly associated with decreased melatonin secretion. However, there was no significant difference between circadian amplitude of the plasma melatonin between healthy elderlies and young adults^13^. Instead of aging, the degree of pineal calcification was associated with melatonin excretion amount^14^.

Substantial evidence suggests genetic factors also play a significant role in melatonin secretion^15,16^. Genome-wide association study (GWAS) has been introduced as a powerful tool to identify common genetic variants of complex diseases or quantitative traits^17^. Currently, there is no published GWAS regarding melatonin levels. Here, we conducted the first GWAS of urine melatonin metabolite, 6-hydroxymelatonin sulfate (aMT6s), which surrogate the circulating melatonin level^18^.

## Materials and methods

### Study population

Five thousand individuals aged 30–70 years old and without cancer history were enrolled from Taiwan Biobank. Biological specimens, personal and clinical information as delinked data were used in this study. Individuals with a record of neurological disorders or psychiatric illnesses were excluded from this study as these conditions may affect melatonin secretion^19,20^. This study was approved by the Institutional Review Board of Chang Gung Medical Foundation and the Institutional Review Board of National Taiwan University Hospital. All subjects have provided written informed consent, and all methods were carried out in accordance with relevant guidelines and regulations.

### Urine aMT6s and creatinine measurement

It is infeasible to draw blood samples from volunteers in the middle of the night for serum melatonin levels. Urinary aMT6s is the major metabolite of melatonin excreted from the kidneys^21^. Thus, measuring morning urine aMT6s level is a practical alternative for serum melatonin level at night^18^. For better correlation, spot urine aMT6s level should be creatinine-corrected to adjust the effect of variable urinary dilution^22^. Urine aMT6s-to-creatinine ratio (UMCR) was calculated from urinary aMT6s divided by urine creatinine level. The concentration of aMT6s was measured in the urine of Taiwan Biobank subjects by an enzyme-linked immunosorbent assay (ELISA) kit using the manufacturer's protocol (Human Melatonin Sulfate ELISA kit, Elab science). No significant cross-reactivity or interference between melatonin sulfate and analogs was observed. All standards via serial dilution were assayed in duplicates. The urine creatinine level was measured using a chemistry analyzer (AU5800, Beckman Coulter) with compensated Jaffe method.

### Genotyping, quality control and imputation

Genotyping with the Axiom-Taiwan Biobank Array Plate (TWB chip; Affymetrix Inc, Santa Clara, California) was performed at the National Center for Genome Medicine of Academia Sinica^23^. We use PLINK (version 1.9), an open-source whole-genome data analysis toolset, for quality control procedures^24^. For SNPs with batch effect, their genotypes were set as missing. SNPs were excluded if missing genotype rate was high (> 5%), minor allele frequency was low (< 1%) or deviated from Hardy–Weinberg equilibrium (*P* value < 10^−5^). Individuals with discordant sex (self-reported sex incongruent to genetic sex, where genetic male or female was defined by X chromosome homozygosity estimate above 0.8 or below 0.2), high missing genotyping rate (> 5%), extreme heterozygosity rate (more than 5 standard deviations away from the mean) or high identity-by-descent score (≥ 0.1875) implying close relatedness were excluded from subsequent analyses. We computed the principal components on a linkage disequilibrium (LD)-pruned (*r*^2^ < 0.2) set of autosomal variants obtained by removing high-LD regions via PLINK. Genotype imputation was carried out with SHAPEIT^25^ and IMPUTE2^26^. We applied1000 Genomes Project Phase 3 East Asian Ancestry as the reference population. For gene annotation, Genome Reference Consortium Human Build 37 was used. Imputed SNPs with low-quality scores (info score^27^ lower than 0.8) were excluded. Indels were removed by using VCFtools^28^.

### Statistical analyses

Age and estimated glomerular filtration rate (eGFR) were expressed as mean and standard deviation. Urine aMT6s and UMCR were expressed as median and interquartile range. Logarithmic transformation of UMCR was done to normalize the data. GWAS analysis was carried out via PLINK v1.9 with an additive genetic model. We applied linear regression for analyzing associations between SNPs and log UMCR. Covariate adjustment in Model 1 included age, sex, and the first ten principal components of ancestry. eGFR was additionally adjusted in Model 2. We used a genome-wide significance threshold of *P* < 5.0 × 10^−8^^29^. Since this threshold is very conservative for small sample size, we set the level for suggestive significance at *P* < 5 × 10^−6^^30,31^. The Manhattan plot and quantile–quantile plot were generated by the qqman R package^32,33^. Regional association plots were made via LocusZoom^34^. The proportion of phenotypic variance explained by SNP was calculated using the following items: effect size estimate of each minor allele on log UMCR, standard error of the effect size, sample size, and minor allele frequency for the SNP^35^. The statistical power of this study was calculated using methods for quantitative GWAS^36^.

### Bioethics statement

This study was approved by the Institutional Review Board of Chang Gung Medical Foundation and the Institutional Review Board of National Taiwan University Hospital. All subjects have provided written informed consent and all methods were carried out in accordance with relevant guidelines and regulations.

## Results

**Figure 1:**
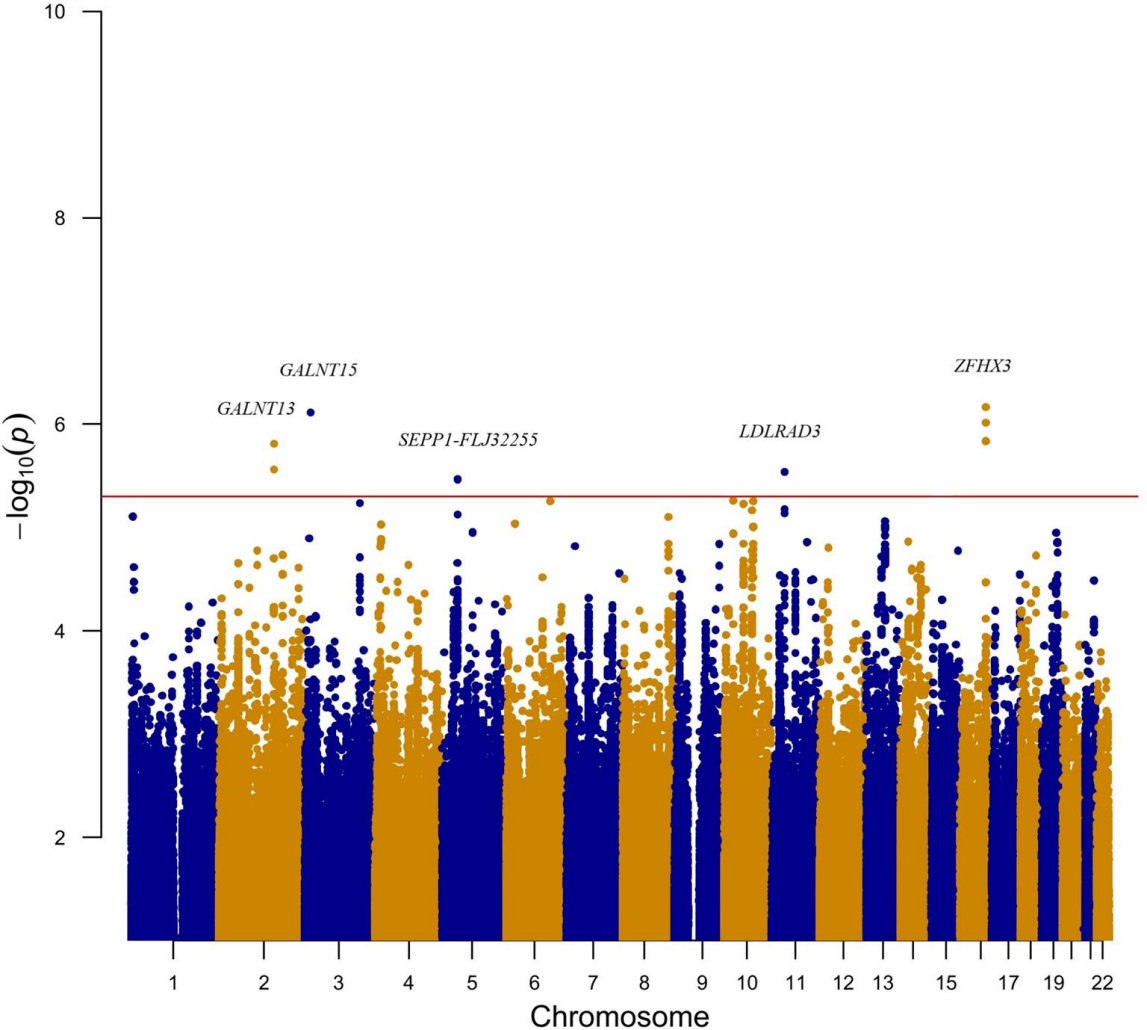
Manhattan plot of the GWAS results for log UMCR. SNPs are plotted on the x axis according to their chromosome position against association with log UMCR on the y axis. The red horizontal line represents the suggestive association threshold of *P* = 5.0 × 10^−6^.

**Figure 2:**
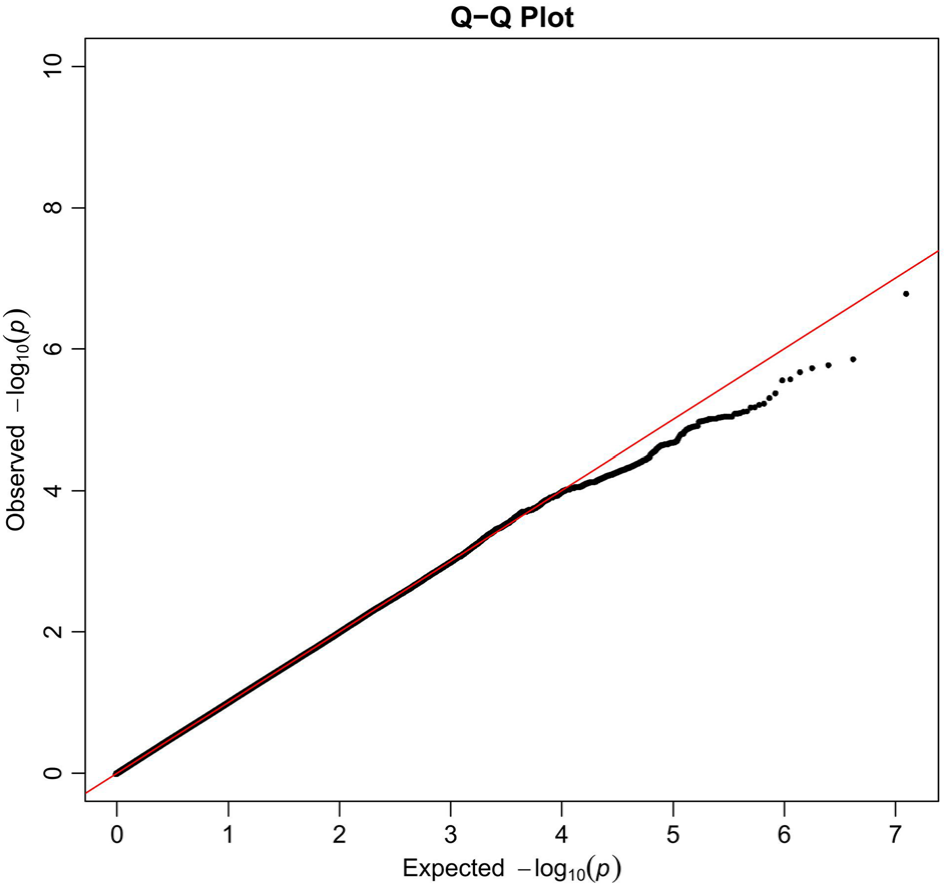
Quantile–quantile plots of log UMCR.

**Figure 3:**
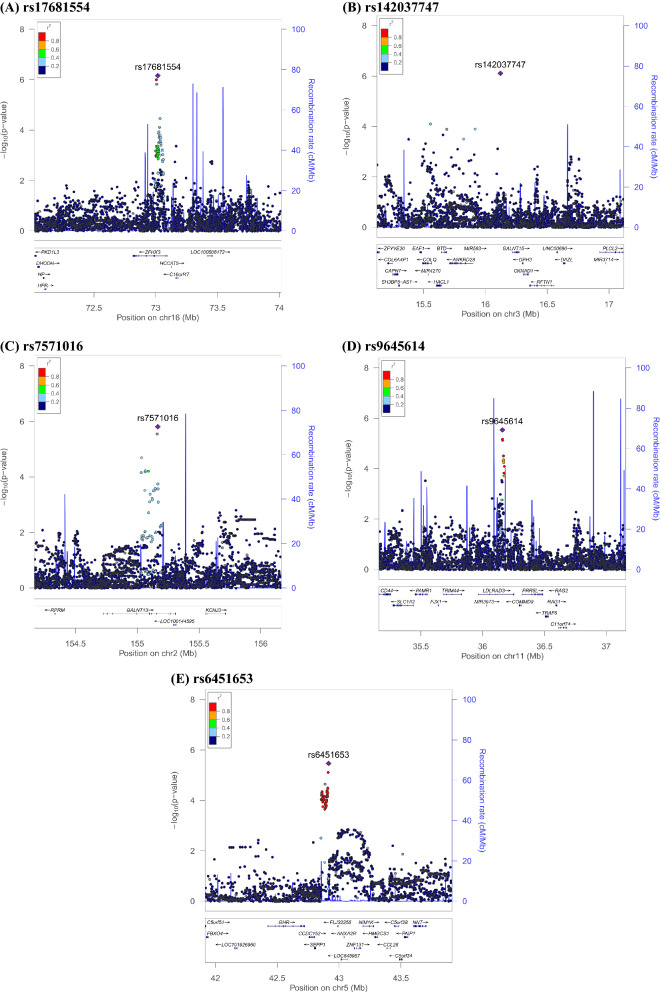
Regional association plots of log UMCR. (**A**) rs17681554, (**B**) rs142037747, (**C**) rs7571016, (**D**) rs9645614, (**E**) rs6451653.

Five thousand subjects were enrolled from Taiwan Biobank initially. One withdrew from the study. 2361 did not have their urine collected in the morning and were excluded. 128 people had documented neurological or psychiatric illness. 137 individuals did not pass quality control procedures. After imputation and quality control, 7,897,704 autosomal SNPs remained. After log transformation of UMCR, data is still not normalized, but the shape of the histogram is better. We performed a GWAS analysis for log UMCR in the remaining 2373 subjects. The characteristics of our study population are listed in Table 1. Age is not significantly associated with log UMCR (Pearson’s r = 0.031; *P* = 0.137). There is no significant gender difference of log UMCR shown by T test (males 1.208, females 1.205; *P* = 0.857). eGFR is also not significantly associated with log UMCR (Pearson’s r = 0.001; *P* = 0.973). Variants with the strongest association in each region regarding to log UMCR are shown in Table 2. Melatonin production is known to be decreased with advanced chronic kidney disease^37^; thus, we adjusted eGFR additionally in Model 2. There was no evidence of liver disorders in these 2373 individuals, and thus no additional model was generated. Figures 1 and 2 are the Manhattan plot and quantile–quantile plot. The genomic inflation factor, defined as the median of the observed chi-squared test statistics divided by the expected median of the corresponding chi-squared distribution, was 1.006. Regional association plots of the top SNPs are shown in Fig. 3. Five loci showed suggestive significance, with one within the *ZFHX3* gene on chromosome 16 (rs17681554; *P* = 6.83 × 10^−7^, Fig. 3a), another near the *GALNT15* gene on chromosome 3 (rs142037747; *P* = 7.82 × 10^−7^, Fig. 3b), a third within the *GALNT13* gene on chromosome 2 (rs7571016; *P* = 1.53 × 10^−6^, Fig. 3c), a fourth within the *LDLRAD3* gene on chromosome 11 (rs9645614; *P* = 2.90 × 10^−6^, Fig. 3d), and the other between *SEPP1* and *FLJ32255* on chromosome 5 (rs6451653; *P* = 3.44 × 10^−6^, Fig. 3e).

**Table 1:** Descriptive characteristics of study subjects.

Characteristics	
Total participants, N	2373
Age, year	50.75 ± 10.83
Males, N (%)	890 (37.51)
eGFR, ml/min/1.73 m^2^	108.20 ± 27.83
Urine aMT6s, ng/ml	20.41 (11.88–30.19)
UMCR, ng/mg	16.98 (10.32–27.33)

Data are mean ± SD, median (IQR) or number (%), as appropriate.

Age is at specimen collection.

*eGFR* estimated glomerular filtration rate (by modification of diet in renal disease equation), *UMCR* urine aMT6s/creatinine ratio.

**Table 2:** Association of genetic loci with log UMCR in a Taiwan Han Chinese population.

SNP	Chr	Position	Nearest gene	UMCR increasing allele	Other allele	UMCR increasing allele frequency	Model 1		Model 2	
							*P* value	Beta (SEM)	*P* value	Beta (SEM)
rs17681554	16	73,016,768	*ZFHX3*	A	C	0.804	6.86 × 10^−7^	0.068 (0.014)	6.83 × 10^−7^	0.068 (0.014)
rs142037747	3	16,121,712	*GALNT15*	G	A	0.989	7.82 × 10^−7^	0.256 (0.052)	7.73 × 10^−7^	0.256 (0.052)
rs7571016	2	155,166,873	*GALNT13*	A	G	0.617	1.53 × 10^−6^	0.056 (0.011)	1.54 × 10^−6^	0.056 (0.012)
rs9645614	11	36,159,947	*LDLRAD3*	A	G	0.953	2.90 × 10^−6^	0.124 (0.026)	2.91 × 10^−6^	0.124 (0.026)
rs6451653	5	42,915,584	*SEPP1-FLJ32255*	G	A	0.869	3.44 × 10^−6^	0.080 (0.017)	3.42 × 10^−6^	0.080 (0.017)

Model 1 was adjusted for age, sex and the first ten principal components of ancestry.

Model 2 was additionally adjusted for eGFR.

*SNP* single nucleotide polymorphism, *Chr* chromosome.

SNPs are imputed with high info score (0.831, 0.988 and 0.946 for rs142037747, rs9645614 and rs6451653, respectively).

Proportion of variance explained by the individual SNPs are 0.98%, 1.01%, 1.08%, 0.95% and 0.92% for rs17681554, rs142037747, rs7571016, rs9645614 and rs6451653 respectively. The calculated power of this GWAS was 56%.

## Discussion

In this first GWAS on melatonin secretion, we identified five suggestive loci associated with variation in log UMCR. rs17681554 is located within *ZFHX3* (Zinc Finger Homeobox 3). ZFHX3 is a transcriptional regulator which contains four homeodomains and seventeen zinc fingers^38^. During neuronal differentiation, there is a preferential expression pattern of ZFHX3 isoforms^39^. In addition, circadian behavior alteration is shown in inducible conditional *Zfhx3* knockout adult mice^40^. Further studies are needed to elucidate a direct linkage between *ZFHX3* and melatonin.

rs142037747 and rs7571016 are located near *GALNT15* (polypeptide N-acetylgalactosaminyltransferase 15) and within *GALNT13* (polypeptide N-acetylgalactosaminyltransferase 13), respectively. These two polypeptide N-acetylgalactosaminyltransferases of the same family catalyze initiation of mucin-type O-linked glycosylation by adding N-acetylgalactosamine to serine or threonine residues of the polypeptide chain^41,42^. Glycosylation is associated with cell adhesion, signal transduction, molecular trafficking, and differentiation in central nervous system development^43^. Whether and how GALNT15 or GALNT13 significantly affects melatonin levels remains determined.

rs9645614 is located within *LDLRAD3* (low density lipoprotein receptor class A domain containing 3). LDLRAD3 alters the proteolysis of amyloid precursor protein and increases the production of amyloid beta-peptide (Aβ)^44^. The primary pathogenesis of Alzheimer's disease (AD) has been attributed to the extracellular aggregation of Aβ^45^. Melatonin levels are altered in AD patients, possibly due to decreased suprachiasmatic nucleus cell number and functional pineal gland volume^46^. Patients with neurodegenerative disorder such as Alzheimer's disease exhibit reduced serum and cerebrospinal fluid melatonin levels comparing to age-matched controls^47,48^. Our present unbiased genetic study, revealing the *LDLRAD3* variant associated with melatonin secretion from pineal gland, provides additional evidence for potential mechanistic explanation in AD patients with altered melatonin levels.

rs6451653 is located between *SEPP1* (selenoprotein P, or SELENOP) and pseudogene *FLJ32255*. SEPP1 serves as a phospholipid hydroperoxide glutathione peroxidase and thus protect the plasma membrane from oxidative damage and is expressed in all brain tissues^49^. SEPP1 is secreted from astrocytes to neurons for prevention of oxidative damage^50^. Several studies demonstrated that *Sepp1* knockout mice displayed cerebellar ataxia, anxiety, impaired spatial memory, and widespread neurodegeneration in various studies^51–55^. Also, deletion of *SEPP1* in dogs resulted in central nervous system atrophy and cerebellar ataxia^56^. It is convincing that the *SEPP1* variant is associated with melatonin levels.

This study also showed borderline significance regarding the positive correlation between age and log UMCR. Since aging causes sarcopenia, the subsequently decreased creatinine excretion from urine increases the substance-to-creatinine ratio. Our results support the current concept that aging itself will not cause a decrease in melatonin secretion or excretion.

There was a concern that aMT6s excretion may be altered when renal function declines. A previous study enrolling 20 elderlies demonstrated that 24-h urine aMT6s was a reliable surrogate for plasma melatonin level, at least among individuals with GFR 24.6 ml/min or above^57^. Our study confirmed that morning UMCR is not significantly correlated with eGFR, and adjusting eGFR in GWAS analysis essentially did not influence the results.

There are limitations to our study. First, it lacks replication of the result in another cohort. We searched in the UK Biobank, but melatonin as phenotype does not exist in the database. Moreover, the sample size is relatively small; thus, for the time being, these SNPs can only be seen as suggestive signals. The statistical power of this GWAS is only 56%, and therefore there are true loci that remain to be identified and validated. Also, there might be individuals receiving medications that can affect melatonin levels but not documented in the Taiwan Biobank data due to the imprecise nature of the questionnaire survey. However, production or selling of melatonin pills is illegal in Taiwan, and thus this important confounding factor may not be significant in our study.

In summary, we have performed the first GWAS regarding melatonin secretion to date. This GWAS identified five highly suggestive genetic loci encompassing genes that demonstrated potential functional connectivity between the genes-associated melatonin level and circadian behavior, neuronal differentiation, cerebellar ataxia, neurodegeneration and Alzheimer's disease. Replication and functional studies of these genetic variations are warranted to understand better the regulation of melatonin secretion and related clinical disorders.

## Data Availability

Individual researchers may request to use the data for specific projects on a collaborative basis. Our data has been submitted to the NHGRI-EBI GWAS Catalog (accession ID: GCST90101875).
